# Reeves et al. Respond to “Harnessing Housing Natural Experiments”

**DOI:** 10.1093/aje/kww054

**Published:** 2016-09-01

**Authors:** Aaron Reeves, Amy Clair, Martin McKee, David Stuckler

We appreciate the interest our study (1), which took advantage of a natural experiment design to better ascertain causality than conventional observational studies could, has generated. In their accompanying commentary, Bentley et al. (2) suggest that misclassification of depression symptoms (e.g., someone with depression reporting that they are not depressed) may explain our findings if misclassification rates changed due to the reduction in the Local Housing Allowance, one component of the United Kingdom's Housing Benefit (HB) for people in the private rental sector.

Misclassification can bias estimated effects in 2 ways. First, if misclassification is nondifferential *over time*, it will dilute associations, tending to underestimate effect sizes. This is likely to result from measurement error. Second, differential misclassification can impact the direction of findings. This can occur, for example, if the reduction in the HB itself leads to changes in how people report symptoms of depression. This is what Bentley et al. suggest may have happened and generated a spurious correlation.

Is there evidence to suggest that HB reductions affected how people reported depression in the Annual Population Survey?

We do not find any. It also seems highly unlikely. The main reason is that, according to one evaluation (3), 85% of those affected by the policy were unaware that they had lost income due to the reduction in the HB. Moreover, 75% of this same sample did not know how the HB was calculated (3). It is possible that the remaining 15% changed how they responded, but it is difficult to imagine why people would change the way they responded to 1 specific survey question because of this administrative change in how the HB is calculated.

Nevertheless, to address this possibility, we further investigated whether changes occurred in people's responses about other subjectively assessed health problems, which could similarly have had misclassification bias but which would not plausibly have been affected by the HB reform. These health outcomes therefore served as “falsification tests” for the possibility of misclassification. The self-reported measures were “chest or breathing problems, asthma, bronchitis”; “severe disfigurement, skin conditions, allergies”; and “other health problems or disabilities” (see our Web Appendix (1), available at http://aje.oxfordjournals.org/). Each is ambiguous, open to subjective interpretation, and therefore possibly also subject to time-varying misclassification; but unlike mental health, none of these are associated with HB reform (Table 1). If the reduction in the HB changed how people responded to surveys about self-reported health problems, then it only affected this single response (depression) to this 1 question (health). Our finding seems much more likely to have been a real mental health effect.

**Table 1.** Estimates of the Difference in Difference for Various Subjective Measures of Health Among Private Renters in the United Kingdom (Matching Model) Between April 2009 and March 2013^a^

Model^b^	Health Problem	Difference-in-Difference Estimate^c^ (After April 2011)
1	Depression	0.011 (0.0038)^d,e^
2	Chest/breathing problems	−0.0047 (0.0038)
3	Allergies	−0.0031 (0.0027)
4	“Other health problems”	−0.0020 (0.0030)

Abbreviation: HB, Housing Benefit.

^a^ Data were obtained from the United Kingdom's Annual Population Survey. The period April 2009–March 2011 (before reform) was compared with the period April 2011–March 2013 (after reform).

^b^ All models included a measure of change over time, the difference between HB recipients and non-HB recipients before April 2011, and the probability of depressive symptoms among non-HB recipients before April 2011. The number of observations was 150,731 in all 4 models.

^c^ Difference in the probability of a person's reporting the specified health problem.

^d^ Values are presented as mean (standard error).

^e^ *P* < 0.01.

Of course, as Bentley et al. note (2), even a strong natural experiment design cannot rule out potential misclassification bias. We agree. Yet, our evidence consistently suggests that any such bias is likely to have been nondifferential with respect to our hypothesis, leading to conservative estimates of the causal effect. Time-varying differential misclassification is always possible, but in this instance it is highly unlikely.
